# Cost-Effective Synthesis of MXene Cadmium Sulfide (CdS) for Heavy Metal Removal

**DOI:** 10.7759/cureus.70872

**Published:** 2024-10-05

**Authors:** Justin Linda, Geetha A, Vasugi Suresh, Balachandran Subramanian, S Menaka

**Affiliations:** 1 Physiology, Saveetha Dental College and Hospitals, Saveetha Institute of Medical and Technical Science, Saveetha University, Chennai, IND

**Keywords:** antibiotic removal, cds, environmental remediation, heavy metal removal, hydrothermal synthesis, mxene, nanomaterials

## Abstract

Background

Environmental contamination resulting from the release of untreated industrial wastewater has emerged as a critical worldwide issue. These effluents frequently have high levels of heavy metals and antibiotics, which are bad for aquatic ecosystems and human health. Oftentimes, conventional wastewater treatment techniques fall short of effectively eliminating these pollutants. Innovative materials that may efficiently absorb or break down contaminants from contaminated water sources are, therefore, desperately needed. Hydrothermally produced MXene cadmium sulfide (CdS) composites have shown great promise as an adsorbent material because of their special qualities, which include high surface area, chemical stability, and customizable surface functions that improve their adsorption capacity for heavy metals and antibiotics alike.

Aim

The aim of this study is to produce MXene-CdS nanoparticles in a cost-effective method for the simultaneous removal of heavy metals from aqueous contaminants for water pollution control.

Methods and materials

MXenes were synthesized by selectively etching Ti_3_AlC_2_ MAX-phase ceramics using aqueous HF. CdS nanoparticles were synthesized separately and integrated with MXenes via a hydrothermal process. The resulting MXene CdS nanocomposites were characterized using scanning electron microscopy (SEM) for morphology, energy dispersion spectrum (EDS) for elemental composition, X-ray diffraction (XRD) study for phase identification, and removal of heavy metals via MXene CdS.

Results

Consistent distribution of CdS nanoparticles on the MXene surface and the creation of MXene CdS nanomembranes with a well-defined shape were observed by SEM analysis. Ti, C, Cd, and S elements, indiciaries of a successful composite formation, were confirmed to be present by EDS. The crystalline structure of both the MXene and CdS phases was confirmed by the distinctive peaks seen in the XRD patterns. MXene-CdS composites facilitate the effective removal of chromium ions from contaminated water. The excellent hydrophilicity of the produced nanomembrane allowed for effective interaction with watery contaminants.

Conclusion

This study showcases the successful synthesis and characterization of MXene-CdS nanocomposites for environmental remediation, particularly in removing toxic metals like chromium from industrial effluents. SEM analysis confirmed the uniform distribution of CdS nanoparticles on the MXene surface, while elemental composition validated their integration. XRD analysis confirmed the crystalline structures of both components. The nanocomposite exhibited excellent hydrophilicity, enhancing the efficient adsorption of heavy metals. Its large surface area and chemical stability contribute to high adsorption efficiency, making it ideal for wastewater treatment. The scalable synthesis process supports practical applications. This research highlights MXene-CdS nanocomposites as a cost-effective, sustainable solution for water pollution control.

## Introduction

Aquatic environments are often contaminated with wastewater containing pollutants like antibiotics and heavy metals from sources such as hospital sewage and soil toxins. These contaminants can promote the exchange of resistant genes among pathogenic bacteria, complicating disease treatment and posing ecological risks. Heavy metals, known for their high toxicity and irreversibility, disrupt plant metabolic functions by affecting ion adsorption and regulation in aquatic environments. Antibiotics, not fully absorbed by organisms, are excreted and found in various water sources, including drinking water. The growing environmental impact of these pollutants highlights the urgent need for effective removal methods to mitigate their toxic effects. MXene is a newly emerging class of two-dimensional inorganic compounds. They are composed of layers so thin their thickness is atomic, of metal carbides, nitrides, or carbonitrides. MXenes possess an inbuilt characteristic of good conductivity as well as appreciable volumetric capacitance owing to the fact that they are synthesized from the carbides and nitrides of transition metals such as titanium. The first titanium carbide MXene, Ti_3_C_2_, was originally discovered and synthesized in 2011 by researchers at Drexel University. They have been receiving attention from researchers due to their graphene-like properties, which include high electrical conductivity, large surface area, and excellent mechanical strength, making them promising materials for a range of applications including energy storage, catalysis, and environmental remediation [1]. MXene has been revealed as a breakthrough material in various disciplines of applications due to its amazing properties. The most widely studied form of MXene is Ti_3_C_2_T_x_. Here, x is the number of terminating groups, and T stands for the O, OH, or/and F groups. Using layered carbide, nitride, or carbonitride precursors, certain atomic layers have been chemically etched to create more than 20 different types of MXenes [2]. MXenes are procured from their corresponding layered MAX phases by removing the A element. In MXenes, the "enes" is suffixed to signify the 2D structure of the material. By virtue of its flexibility, structural stability, hydrophilic surfaces, and electrical conductivity, Ti_3_C_2_T_x_ has applications in supercapacitors, nanofiltration membranes, oxygen evolution reactions, etc. [3]. 

The allure of MXenes arises due to their unmatched characteristics, like a good surface-to-volume ratio, impressive electric conductivity, and adsorption in the near-infrared region, along with the lack of difficulty associated with functionalizing MXene surfaces with diverse polymers or nanoparticles. MXenes are also largely effective as adsorbents. They can serve as a good adsorbent for heavy metal pollutants such as lead, copper, chromium, mercury, and barium, as well as pharmaceutical compounds. There are, however, certain roadblocks that hinder the commercial utilization of MXene for this purpose [4]. Some of these are the problems of the resilience of MXenes and in finding less toxic and cost-effective methods of MXene synthesis. MXenes have the ability to absorb various ionic and molecular species due to their hydrophobic nature and the energetic chemical groups present on their surface. This capability can be utilized for environmental pollution control as well as environmental monitoring. MXene can be used in membrane separation, adsorption, and photocatalysis, among other separation processes. As a versatile adsorbent, MXene can effectively remove hazardous pollutants from the environment, including dyes, toxic chemicals, and hazardous metals like uranium and thorium. MXenes retain antimicrobial properties, and hence they are found in filter membranes [5].

A great deal of pharmaceutically active compounds have been discharged into the environment with no proper treatment over the last couple of decades. Antibiotics are a very dangerous class of these compounds and have adverse effects on aquatic environments. Similarly, harmful toxins like heavy metals tend to bioaccumulate and then slowly release into waterways, risking aquatic life. MXene materials show remarkable capability in photocatalytic degradation of different antibiotics. MXene adsorbents were revealed to show impressive adsorption capacity for the successful elimination of ions containing metallic elements from a water-based solution [6]. The frequency of hazardous pollutants, such as dyes, heavy metals, and antibiotics found in effluents, has increased in the last few decades. Currently, techniques on the energy and environmental use of MXene-based nanomaterials are mainly in photocatalysis, adsorption, and water splitting. MXene adsorbents and photocatalysts have been widely used for successful remediation of pollutants like dyes and wastewater with metal ions [7]. MXenes have been found to be capable of adsorbing several heavy metals, such as lead, copper, chromium, and mercury. Their surface groups interact with the target pollutants to adsorb them [8]. Cancer can result from radioactive substances and toxic substances like arsenic being present. The identification of these poisons is crucial. MXenes have emerged as valuable materials for sensing and adsorbing heavy metal ions. The toxicity in relation to the bioaccumulation of metals like cadmium, nickel, and arsenic can vary from kidney and bladder diseases to nervous system disorders and even carcinogenic potential. Mercury traces in live tissues can lead to neurological damage, chromosomal mutations, and others [9].

Several treatment approaches are currently being studied to remove metallic ions and liquid waste, including ion exchange, adsorption, membrane filtration, chemical precipitation, and electrochemical coagulation. Among these methods, adsorption has been found to be the most economical, efficient, environmentally friendly, and user-friendly technique. MXenes are used as adsorbents for the removal of heavy metals from water. In the process of heavy metal removal through adsorption, MXene-based composites have demonstrated strong elimination capabilities, rapid optimal efficiency, and superior specific capacity [10]. Cadmium Sulfide (CdS) is a yellow to orange-colored, crystalline, inorganic compound. CdS is a naturally occurring sulfide as seen in minerals like greenockite and hawleyite and is also produced by sulfate-reducing bacteria. Colorants, polymers, fabrics, transparent materials, and ceramics all employ CdS as a color. Photovoltaic cells, radon and fire detectors, luminescent diodes, and other devices all use CdS. CdS is synthesized by a number of methods, like microwave-assisted precipitation, the wet chemical method, microwave irradiation, gamma irradiation, biological methods, and mechanochemical methods. The last method is also an environmentally friendly method since no harmful solvents are used [11]. 

Antibiotics are extensively utilized for the treatment of bacterial infections. Despite their benefits, they also easily enter the environment and ecosystem and pollute them. A study using a one-pot solvothermal approach demonstrated improved photocatalytic antibiotic elimination efficiency and excellent stability in the synthesis of zinc-doped CdS nanostructures (Zn-CdSC) coated with core-shell graphene. Zn-CdSC nanostructures were found to exhibit exceptional performance in the photocatalytic process used to remove metal ions [12]. A composite material consisting of CdS-MWCNTs (multiwalled carbon nanotubes) was employed as an adsorbent to remove antibiotics, namely cefotaxime, cefradine, and cefazolin. It was discovered that the CdS-MWCNT nanocomposites had a significant adsorption capacity and a high specific surface area. So it is evident that CdS is being used in composites synthesized for antibiotic removal. In a study conducted, an S-scheme Bismuth oxychloride/CdS nanoparticles were synthesized. The tetracycline hydrochloride (TC-HCl) antibiotic was used as the model pollutant to estimate the photocatalytic properties of BiOCl/CdS nanocomposite. The nanocomposite showed enhanced photocatalytic removal of TC-HCl [13]. A CdS nanoparticle-interspersed-Bi_2_MoO_6_ heterojunction photocatalyst was fabricated. It was observed that the CdS nanocomposite showed enhanced visible light photocatalytic activity for the removal of the levofloxacin antibiotic, which was used as the model pollutant. Previously conducted research highlighted that a nanocomposite of CdS/BiOBr heterojunction photocatalyst was fabricated, and it was demonstrated that the nanocomposite shows great potential for the degradation of toxic antibiotics presented in water [14]. Using a hydrothermal process, CdS semiconductor photocatalysts were easily fabricated without the need for capping, organic solvent, or surfactant. It was found that the degradation of azo dye and ofloxacin antibiotic was performed at 98% and 88%, respectively, in the presence of sunshine. The contaminants' photodegradation indicates that the created CdS photocatalyst has the potential to be an effective, reusable material that functions as a sunlight-active photodegradation substance for environmental cleaning and protection. A polypyrrole/CdS/nickel hollow fiber bearing photocatalytic activity was manufactured, and its pollutant removal efficacy was assessed and found to be significant. This novel material showed good photocatalytic stability and recyclability as well. Another experiment synthesized a compound of ceric dioxide modified with CdS. It was prepared via a one-step hydrothermal method. It was observed that 90% of ciprofloxacin (the model antibiotic pollutant used) can be photodegraded by the CeO_2_/CdS/RGO composite material. The material was seen to possess appreciable photocatalytic activity on various pollutants. The modification of CeO_2_ with CdS was shown to vastly improve its photocatalytic activity. It was shown that a composite synthesizer of graphite carbon nitride and CdS functionalized with carboxylic acid exhibited strong photocatalytic activity for the breakdown of the antibiotic sulfamethazine [15].

We synthesized a novel 2D nanosheet composite of Ti_3_C_2_ MXene with CdS nanoflowers, which exhibited excellent photocatalytic properties. The photocatalytic activity of CdS was significantly enhanced by its integration with MXene, making this material promising for environmental applications such as pollutant removal. MXene-based composites demonstrated superior adsorption capabilities for dyes, eutrophic chemicals, and heavy metal ions, indicating their potential as effective adsorbents for water remediation. This study explores the synthesis, properties, and potential applications of MXenes and CdS, highlighting their benefits in environmental and ecological sustainability, particularly in water purification and related fields.

## Materials and methods

MXene synthesis (Ti₃C₂F₂-MXene)

The synthesis of Ti_3_C2F_2_-MXene begins with the use of Ti_3_AlC_2_, commonly known as titanium aluminum carbide, as the precursor material. A ratio of 1:1 for Ti_3_AlC_2_ and LiF_3_ is maintained, with 30 mL of 6 M hydrochloric acid (HCl) used in the process. The Ti_3_AlC_2_, HCl, and LiF_3_ are mixed together, and the solution is stirred continuously at 90°C for a duration of two hours. During this time, the aluminum layers in Ti_3_AlC_2_ are selectively etched away by the acid, aided by the fluoride ions from LiF_3_. This process results in the formation of Ti_3_C_2_F_2_-MXene, a 2D material with a layered structure. The resulting product is referred to as Solution A, which contains the synthesized MXene in a liquid phase.

CdS synthesis

CdS was synthesized using a simple precipitation method. The process begins by dissolving 0.1 M of cadmium nitrate in 100 mL of water, a step that ensures the cadmium ions are adequately dispersed in the solution. The solution is stirred for 30 minutes at 25°C to facilitate complete dissolution and homogeneity. Once the cadmium nitrate is fully dissolved, sodium sulfide is added dropwise to the solution. This step leads to the formation of CdS as a yellow precipitate. The color change indicates the successful formation of CdS, which is a semiconductor material. The resulting suspension, referred to as Solution B, contains the CdS in a fine precipitate form.

Formation of composite material

To create the composite material, Solution B, containing CdS, is added to Solution A, which contains the synthesized MXene. The two solutions are mixed together and stirred for two hours to ensure thorough dispersion and interaction between the CdS particles and the MXene layers. Following this mixing step, the solution undergoes hydrothermal treatment; the reaction mixture is transferred to a hydrothermal reactor and heated to 180°C for 12 hours. This process promotes crystallization and the formation of a well-structured composite material. After the hydrothermal treatment, the reaction product is filtered to remove any unreacted components. The filtered material is washed sequentially with water and ethanol to remove impurities. Finally, the material is dried at 80°C for 12 hours to obtain a solid powder of the MXene-CdS composite. The structural, morphological, and compositional characteristics of the synthesized material are analyzed using a range of advanced techniques, such as X-ray diffraction (XRD) analysis with a Cu source, scanning electron microscopy (SEM), and energy dispersive spectroscopy (EDS). Sample preparation for SEM analysis involves mounting the dried composite powder onto a conductive adhesive stub and gold sputtering to enhance conductivity.

Removal of heavy metals via MXene CdS

The removal of heavy metals, specifically chromium ions, using MXene materials involves a methodology that promotes the adsorption and reduction of these ions. MXenes effectively interact with chromium, enhancing its removal from contaminated sources. The process is further optimized through irradiation techniques; solar light irradiation boosts the photocatalytic activity, significantly improving removal efficiency, while UV light irradiation provides additional energy to accelerate the reduction process. Together, these methods facilitate the effective reduction of chromium ion concentrations in contaminated water, utilizing sustainable light sources for enhanced efficacy.

## Results

XRD analysis of MXene CdS

**Figure 1:**
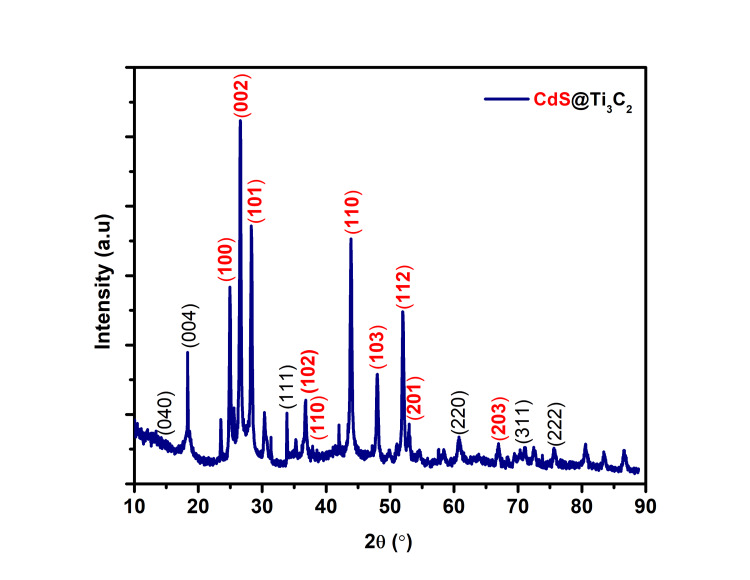
XRD pattern of MXene CdS XRD, X-ray diffraction study; CdS, cadmium sulfate

XRD analysis is used for characterizing the crystal structure of the synthesized MXene-CdS composite material. MXene CdS typically exhibits a layered structure, and XRD provides insight into interlayer spacing and crystallographic orientation. To obtain the XRD pattern, the composite sample is exposed to X-ray radiation, and the scattered intensity is measured at various angles. The resulting XRD pattern features multiple peaks, each corresponding to diffraction from different crystal planes. Notably, the (002) peak is the most commonly observed, indicating the interlayer spacing of the MXene-CdS layers, while its position and intensity can reveal information about the thickness of the MXene-CdS layers and the degree of exfoliation. Additional peaks such as (100), (101), (110), and (103) may also be present, depending on the specific composition and crystal structure of the MXene-CdS material (Figure 1). These peaks facilitate further analysis of the crystallographic orientation and quality of the composite. The XRD patterns determine that the samples are composed of Mxene (JCPDS 52-0875) CdS (JCPDS No. 77-2306) material. It is important to note that the precise positions and intensities of the XRD peaks can vary based on factors like the synthesis method of the MXene-CdS composite, surface functionalization, and the presence of impurities.

SEM analysis of MXene CdS

**Figure 2:**
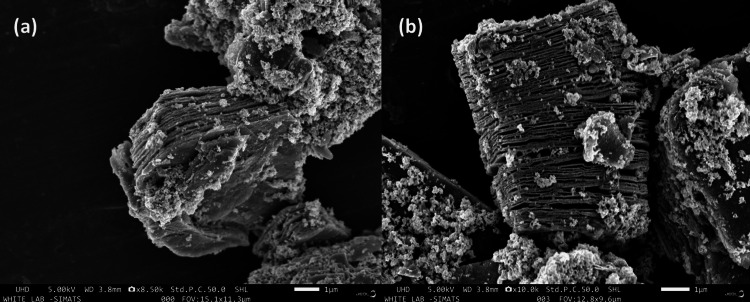
SEM analysis of MXene CdS: a) 8.5k resolution and b) 10k resolution XRD, X-ray diffraction study; CdS, cadmium sulfate

Figure 2 shows the SEM analysis for MXene CdS. SEM analysis is commonly used to examine the surface morphology and microstructure of MXene materials. The SEM images provide valuable insights into the shape, size, and distribution of MXene nanosheets or particles. SEM images captured the presence of individual MXene nanosheets or interconnected networks of nanosheets, providing insights into the exfoliation and delamination processes during MXene synthesis.

Surface Topography

SEM images allowed the observation of surface roughness and variations in the MXene material. The analysis can reveal the presence of surface defects, cracks, or irregularities, which may impact the material's properties and potential applications. Agglomeration can impact the material's properties and hinder its performance in applications, whereas good dispersion leads to improved accessibility and interactions with other materials or analytes. Ti_3_C_2_ MXene typically exhibits a layered or sheet-like structure. The individual layers are stacked on top of each other to form a multilayer structure.

Surface Roughness

The surface of Ti_3_C_2_ sheets can exhibit varying degrees of roughness. This roughness can arise from the presence of residual functional groups, surface defects, or the formation of wrinkles or folds during the delamination process.

Intercalation of Solvents or Functional Molecules

Ti_3_C_2_ MXene has a hydrophilic surface that can readily interact with various solvents or functional molecules. This allows for the intercalation or adsorption of molecules on the surface, leading to changes in the surface morphology and properties.

EDS analysis of MXene CdS

**Figure 3:**
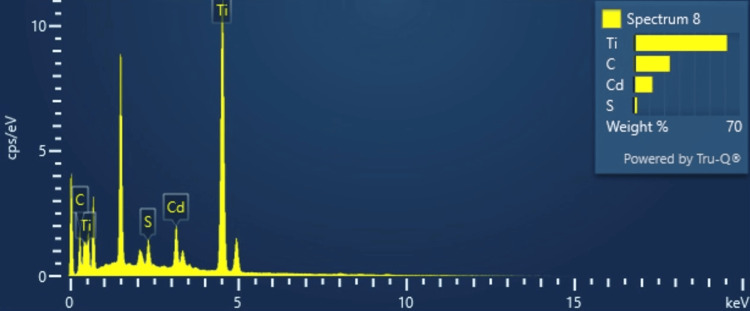
EDS analysis of Mxene CdS EDS, energy dispersion spectrum; CdS, cadmium sulfate

Figure 3 shows that the elemental composition EDS analysis can provide elemental composition information of materials, including Ti_3_C_2_ (a type of MXene).

Elemental Composition

EDS analysis can identify and quantify the elements present in the Ti_3_C_2_ sample. The primary constituents of Ti_3_C_2_ are titanium (Ti) and carbon (C). The EDS spectrum displays characteristic X-ray peaks corresponding to these elements. The intensities of these peaks can be used to determine the relative abundance of each element.

Stoichiometry

EDS analysis helps confirm the stoichiometric ratio of titanium and carbon in Ti_3_C_2_. By comparing the intensities of the titanium and carbon peaks in the EDS spectrum, the atomic ratio of Ti to C can be calculated. This information is essential for verifying the composition and structure of Ti_3_C_2_.

Impurity Identification

EDS analysis can detect the presence of impurities or trace elements in the Ti_3_C_2_ sample. It can identify additional elements that may be present due to impurities in the starting materials or synthesis process. The EDS spectrum may exhibit peaks corresponding to these impurity elements, providing insights into the purity of the Ti_3_C_2_ material.

Mapping

EDS mapping can be performed to visualize the spatial distribution of elements within the Ti_3_C_2_ sample. By acquiring EDS spectra at multiple points across the sample and correlating the X-ray intensities with their spatial locations, elemental maps can be generated. EDS mapping of Ti_3_C_2_ can provide information about the distribution and homogeneity of titanium and carbon within the material.

Removal of heavy metals via MXene CdS

**Figure 4:**
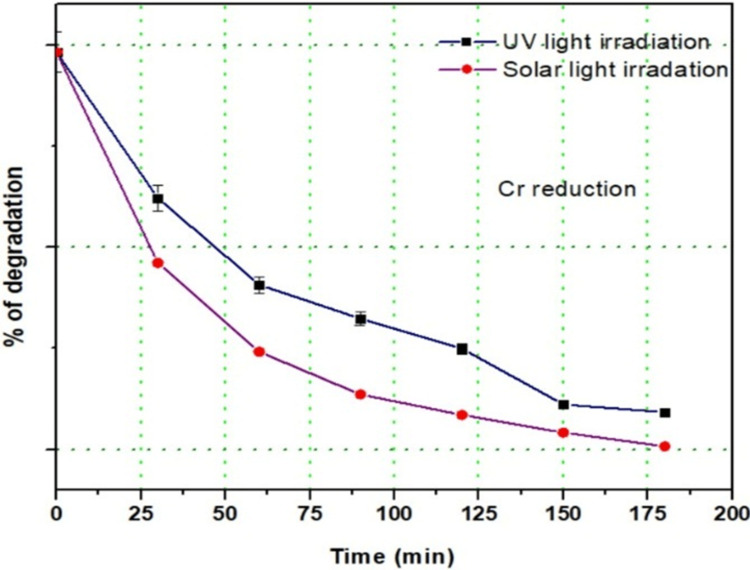
Heavy metal removal via MXene CdS CdS, cadmium sulfate

When exposed to solar and UV light, the MXene composites facilitate the effective removal of chromium ions from contaminated water. The MXenes interact with chromium, promoting adsorption and reduction. The presence of reactive oxygen species generated by the photocatalytic activity enhances the degradation of chromium ions, preventing the recombination of photoelectron-hole pairs. This method, optimized through both solar and UV light irradiation, allows for significant improvements in removal efficiency (Figure 4). As demonstrated, the system achieved a degradation efficiency of 94% after three cycles, indicating its effectiveness even after repeated use. This technology presents a promising solution for addressing chromium contamination in water and offers a versatile approach to tackling various water pollution issues.

## Discussion

MXene materials, like CdS-modified MXenes, are widely researched for their promising properties in removing antibiotics and heavy metals. Figure 1 shows the XRD for MXene. The most commonly observed peak is the (002) peak, which corresponds to the interlayer spacing of the MXene layers. These peaks can be used to further analyze the crystallographic orientation and quality of the MXene material. It is important to note that the exact positions and intensities of the XRD peaks can vary depending on factors such as the specific MXene synthesis method, surface functionalization, and the presence of impurities. The XRD pattern of the CdS Ti_3_C_2_ composite shows no change in the characteristic peaks of CdS and the diffraction peaks corresponding to the (002) and (004) planes of Ti_3_C_2_, indicating that the crystal structures of CdS and Ti_3_C_2_ MXene do not interact. This might imply that, rather than a shift in the crystal structure of CdS, the significant increase in photocatalytic activity is due to the co-catalytic action of Ti_3_C_2_ as a growth substrate [16].

SEM is employed to analyze the surface morphology and microstructure of MXene CdS materials. Figure 2 shows the SEM analysis for MXene CdS. SEM analysis is commonly used to examine the surface morphology and microstructure of MXene materials. The SEM images provide valuable insights into the shape, size, and distribution of MXene nanosheets or particles. When discussing the SEM results of MXene, the following are some key points to consider.

Morphology and structure

SEM images reveal the overall morphology of the MXene material, whether it is in the form of layered nanosheets, hierarchical structures, or agglomerated particles [17]. The SEM analysis can show the presence of wrinkles, folds, or crumpled structures on the MXene surface, which are characteristic features of MXene materials.

Sheet-like structure

MXene materials typically exhibit a sheet-like structure with a large lateral size. SEM images captured the presence of individual MXene nanosheets or interconnected networks of nanosheets, providing insights into the exfoliation and delamination processes during MXene synthesis.

Surface topography

SEM images allowed the observation of surface roughness and variations in the MXene material [18]. The analysis can reveal the presence of surface defects, cracks, or irregularities, which may impact the material's properties and potential applications.

Particle size and distribution

If MXene is synthesized in the form of particles or flakes, SEM images can provide information about their size and distribution. Particle size distribution analysis can be performed based on the SEM images, enabling quantitative characterization of the MXene material.

Agglomeration and dispersion

SEM images can indicate the extent of agglomeration or dispersion of MXene particles or nanosheets [19]. Agglomeration can impact the material's properties and hinder its performance in applications, whereas good dispersion leads to improved accessibility and interactions with other materials or analytes.

Comparative analysis

SEM images can be used for comparative analysis between different MXene samples, synthesis methods, or surface modifications. By comparing SEM images, researchers can identify differences in morphology, structure, or dispersion, providing insights into the effects of various parameters on the MXene material [20]. Ti_3_C_2_ MXene typically exhibits a layered or sheet-like structure. The individual layers are stacked on top of each other to form a multilayer structure.

Delaminated sheets

The synthesis process of Ti_3_C_2_ involves the selective etching of an aluminum (Al) layer from a precursor MAX phase material. This results in the delamination of the layers, leading to the formation of Ti_3_C_2_ sheets with a large surface area [21].

Surface roughness

The surface of Ti_3_C_2_ sheets can exhibit varying degrees of roughness. This roughness can arise from the presence of residual functional groups, surface defects, or the formation of wrinkles or folds during the delamination process.

Intercalation of solvents or functional molecules

Ti_3_C_2_ MXene has a hydrophilic surface that can readily interact with various solvents or functional molecules. This allows for the intercalation or adsorption of molecules on the surface, leading to changes in the surface morphology and properties [22].

EDS provides elemental composition data by detecting X-rays emitted from a sample when it is bombarded with electrons. This technique identifies and quantifies the elements present in a MXene CdS material. Elemental composition EDS analysis can provide elemental composition information of materials, including Ti_3_C_2_ (a type of MXene) [23].

Elemental composition

EDS analysis can identify and quantify the elements present in the Ti_3_C_2_ sample. The primary constituents of Ti_3_C_2_ are titanium (Ti) and carbon (C). The EDS spectrum displays characteristic X-ray peaks corresponding to these elements. The intensities of these peaks can be used to determine the relative abundance of each element [24].

Stoichiometry

EDS analysis helps confirm the stoichiometric ratio of titanium and carbon in Ti_3_C_2_. By comparing the intensities of the titanium and carbon peaks in the EDS spectrum, the atomic ratio of Ti to C can be calculated. This information is essential for verifying the composition and structure of Ti_3_C_2_.

Impurity identification

EDS analysis can detect the presence of impurities or trace elements in the Ti_3_C_2_ sample [25]. It can identify additional elements that may be present due to impurities in the starting materials or synthesis process. The EDS spectrum may exhibit peaks corresponding to these impurity elements, providing insights into the purity of the Ti_3_C_2_ material.

Mapping

EDS mapping can be performed to visualize the spatial distribution of elements within the Ti_3_C_2_ sample [26]. By acquiring EDS spectra at multiple points across the sample and correlating the X-ray intensities with their spatial locations, elemental maps can be generated. EDS mapping of Ti_3_C_2_ can provide information about the distribution and homogeneity of titanium and carbon within the material [27].

In a study, a new Ti_2_CT_x_ MXene was created by ultrasonic exfoliation after the Al layer of Ti_2_AlC was etched. This compound was subsequently used to remove methylene blue (MB) and heavy metals like chromium (Cr). The cationic dye MB was used as the model pollutant. We carefully examined the effects of temperature, pH, background ions, contact duration, and starting MB concentration on the adsorption process of MB. The synthesized MXene was shown to have exceptional adsorption characteristics for the elimination of cationic dye (MB), with a maximum adsorption capacity of 2460.9 mg g-1 [28]. In a study performed, a novel two-dimensional material, an urchin-like rutile TiO_2_-C/TiC was prepared. The self-assembling two-dimensional MXene derivative showed outstanding chemical activities in environmental applications and was observed to be capable of adsorbing heavy metal Cr (IV) in all forms. MXene also displays a good adsorption ability by electrostatic adsorption and chemical adsorption. Comparing pure Ti_3_C_2_ nanosheets to the TiO_2_/Ti_3_C_2_/TiO_2_ composite material, the study's findings demonstrated a much-improved capacity to remove harmful Cr (VI) from water, with 99.35% of Cr (VI) effectively removed [29]. High catalytic activity was demonstrated by a novel ternary nanocomposite, MXene-Au-CdS, which was developed and used in the field of photocatalytic hydrogen generation. MXene-Au-CdS has a hydrogen generation rate of 17,070.43 μmol g-1 h-1, 1.85 times that of CdS under visible light irradiation [30].

Limitation

The limitations of the study include the need for further research to evaluate the long-term stability and reusability of the MXene-CdS nanocomposites. Additionally, the scalability of the synthesis process for large-scale industrial applications has not been fully explored. The study also lacks a comprehensive assessment of the potential environmental impacts and toxicity of the nanocomposites themselves, which could be crucial for real-world implementation. Finally, the efficiency of the material in varying environmental conditions and against a broader range of contaminants remains to be investigated.

## Conclusions

This study demonstrates the successful synthesis and characterization of MXene-CdS nanocomposites, emphasizing their potential for environmental remediation, particularly for the removal of toxic metals such as chromium from industrial effluents. SEM analysis confirmed the uniform distribution of CdS nanoparticles on the MXene surface, while elemental composition verified the successful integration of CdS with MXenes. XRD analysis confirmed the crystalline structures of both MXene and CdS. The nanomembrane exhibited excellent hydrophilicity, promoting efficient adsorption of heavy metals, including chromium, from aqueous solutions. Furthermore, the large surface area and chemical stability of the composite contribute to its high adsorption efficiency, making it ideal for environmental applications. The scalability of the synthesis process supports its practical application in industrial wastewater treatment systems. This study highlights the potential of MXene-CdS nanocomposites as a cost-effective, sustainable solution for water pollution control. Future research should aim at optimizing the production process and evaluating the composite’s performance under diverse environmental conditions to maximize its potential for large-scale remediation.

## References

[1] Das Chakraborty S, Kumar U, Bhattacharya P, Mishra T (2024). Tailoring of visible to near-infrared active 2D MXene with defect-enriched titania-based heterojunction photocatalyst for green H(2) generation. ACS Appl Mater Interfaces.

[2] Rathina Gesav VR, Geetha A, Vasugi S, Balachandran S, Ilangovar IG (2024). Emerging two-dimensional Ti3C2-BiOCl nanoparticles for excellent antimicrobial and antioxidant properties. Cureus.

[3] Hai T, Basem A, Alizadeh A (2024). Optimizing Gaussian process regression (GPR) hyperparameters with three metaheuristic algorithms for viscosity prediction of suspensions containing microencapsulated PCMs. Sci Rep.

[4] Kumar JA, Prakash P, Krithiga T (2022). Methods of synthesis, characteristics, and environmental applications of MXene: a comprehensive review. Chemosphere.

[5] Lim GP, Soon CF, Ma NL, Morsin M, Nayan N, Ahmad MK, Tee KS (2021). Cytotoxicity of MXene-based nanomaterials for biomedical applications: a mini review. Environ Res.

[6] Farooque AJ, Ihsanullah I, Muhammad B, Roberto CM, Grzegorz B, Fausto G (2023). MXene-based materials for removal of antibiotics and heavy metals from wastewater - a review. Water Resour Ind.

[7] Saafie N, Zulfiqar M, Samsudin MFR, Sufian S (2022). Current scenario of mxene-based nanomaterials for wastewater remediation: a review. Chemistry.

[8] Kashif R, Ravi PP, Abdul R, Samantha B, Yury G, Khaled AM (2019). Water treatment and environmental remediation applications of two-dimensional metal carbides (MXenes). Mater Today.

[9] Sikaria S, Celshia S, Selvamani M, Suresh V, Hussein MA (2024). Electrochemical detection of ascorbic acid by fe₂o₃ nanoparticles modified glassy carbon electrode. Cureus.

[10] Othman Z, Mackey HR, Mahmoud KA (2022). A critical overview of MXenes adsorption behavior toward heavy metals. Chemosphere.

[11] Tunesi MM, Soomro RA, Han X, Zhu Q, Wei Y, Xu B (2021). Application of MXenes in environmental remediation technologies. Nano Converg.

[12] Krisha SG, Menaka S, Celshia S, Selvamani M, Suresh V (2024). Synthesis of copper molybdate and its electrochemical sensing of paracetamol. Cureus.

[13] Liu T, Wang B, Wang T, Li C, Wang W, Wang M, Zhang J (2022). One-pot synthesis of Zn-CdS@C nanoarchitecture with improved photocatalytic performance toward antibiotic degradation. Chemosphere.

[14] Ali F, Sahar R, Mohammad A, Inderjeet T, Shilpi A, Vinod KG (2016). Dynamic adsorption behavior and mechanism of cefotaxime, cefradine and cefazolin antibiotics on CdS-MWCNT nanocomposites. J Mol Liq.

[15] Yang L, Wang J, Zhang Y, Zhou B, Tan P, Pan J (2022). Construction of s-scheme BiOCl/CdS composite for enhanced photocatalytic degradation of antibiotic. J Mater Sci: Mater Electron.

[16] Li S, Liu Y, Long Y, Mo L, Zhang H, Liu J (2018). Facile synthesis of bi2moo6 microspheres decorated by cds nanoparticles with efficient photocatalytic removal of levfloxacin antibiotic. Catalysts.

[17] Teeradech S, Supinya N, Sawitree J, Narong C, Suwat N (2021). CdS/BiOBr heterojunction photocatalyst with high performance for solar-light-driven degradation of ciprofloxacin and norfloxacin antibiotics. Appl Surf Sci.

[18] Anvitha NL, Geetha A, Vasugi S, Balachandran S, Ilangovar IG, Suresh V, Subramanian B (2024). Facile fabrication of titanium carbide (Ti3C2)-bismuth vanadate (BiVO4) nano-coupled oxides for anti-cancer activity. Cureus.

[19] Hongwei P, Jian Z, Shujie Z (2022). Polypyrrole/cadmium sulfide/nickel hollow fiber as an enhanced and recyclable intrinsic photocatalyst for pollutant removal and high-effective hydrogen evolution. Int J Hydrogen Energy.

[20] Yao J, Gao Z, Meng Q, He G, Chen H (2021). One-step synthesis of reduced graphene oxide based ceric dioxide modified with cadmium sulfide (CeO(2)/CdS/RGO) heterojunction with enhanced sunlight-driven photocatalytic activity. J Colloid Interface Sci.

[21] Shihai C, Zhongyi J, Huan C, Fang J, Xin W (2018). Carboxylic acid-functionalized cadmium sulfide/graphitic carbon nitride composite photocatalyst with well-combined interface for sulfamethazine degradation. J Photochem Photobiol A Chem.

[22] Devanshi C, Geetha A, Ilangovar IG, Vasugi S, Balachandran S, Suresh V, Subramanian B (2024). Graphene-functionalized titanium carbide synthesis and characterization and its cytotoxic effect on cancer cell lines. Cureus.

[23] Fan Z, Hua-QZ Hua-QZ, Jie S, Yu-LM Yu-LM, Yun-XP Yun-XP, Shu-HY Shu-HY (2018). Coupling cobalt sulfide nanosheets with cadmium sulfide nanoparticles for highly efficient visible-light-driven photocatalysis. Appl Catal B.

[24] Cao Y, Zhang H, Yin Y, Ge B, Ren G, Shao X (2021). Fabrication of visible-light response cadmium sulfide modified superhydrophobic surface for water resource remediation. Nanotechnology.

[25] Benteng S, Pengyuan Q, Zhangqian L (2021). The fabrication of 1d/2d cds nanorod@ti3c2 mxene composites for good photocatalytic activity of hydrogen generation and ammonia synthesis. Chem Eng J.

[26] Bin S, Xusheng D, Huapeng L (2021). Surface charge engineering for two-dimensional ti2ctx mxene for highly efficient and selective removal of cationic dye from aqueous solution. Sep Purif Technol.

[27] Zou G, Guo J, Peng Q, Zhou A, Zhang Q, Liu B (2016). Synthesis of urchin-like rutile titania carbon nanocomposites by iron-facilitated phase transformation of MXene for environmental remediation. J Mater Chem A Mater Energy Sustain.

[28] Ijaz I, Bukhari A, Nazir A (2023). Functionalization of MXene using iota-carrageenan, maleic anhydride, and N,N'-methylene bis-acrylamide for high-performance removal of thorium (IV), uranium (IV), sulfamethoxazole, and levofloxacin. Int J Biol Macromol.

[29] Wang H, Cui H, Song X, Xu R, Wei N, Tian J, Niu H (2020). Facile synthesis of heterojunction of MXenes/TiO(2) nanoparticles towards enhanced hexavalent chromium removal. J Colloid Interface Sci.

[30] Yin J, Zhan F, Jiao T (2020). Facile preparation of self-assembled mxene@au@cds nanocomposite with enhanced photocatalytic hydrogen production activity. Sci China Mater.

